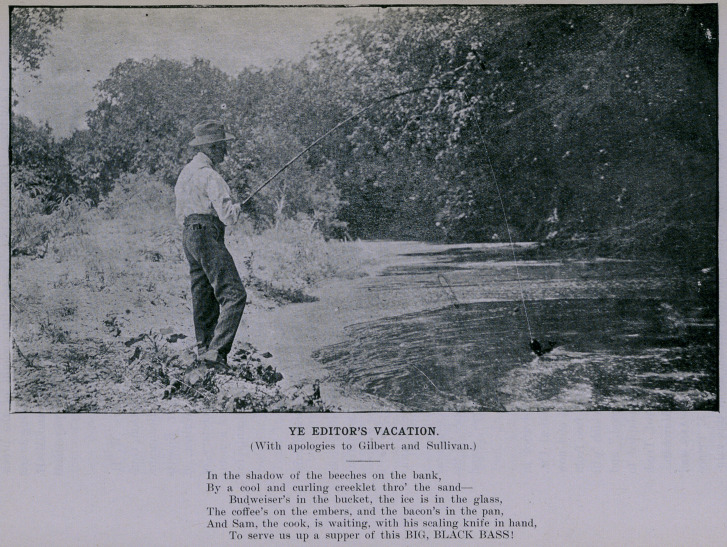# Editorialets

**Published:** 1907-09

**Authors:** 


### Editorialets.

---

General Anesthesia by the Hypodermic Method.—The rapidity with which the Abbott-Lanphear method of anesthesia has advanced in the confidence of the profession is unparalled. The method is simple, easily used, requires less assistants in surgical operations, is acceptable to the patient, is remarkably free from danger, is devoid of after effects to a greater extent than inhalation anesthesia, is recovered from promptly and can be adjusted to nearly all patients. Its most marked influence is in the slowing of the respiration, which is apt to alarm those who have not proven that no harm results, the respiration being that of deep sleep. The use of hyoscine instead of scopolamine is a great improvement on the original method, and the introduction of this substance by Dr. Abbott and of cactus in the form of cactin in the compound hyoscine, morphine and cactin comp. (H. M. C. Abbott), has added a safeguard which is invaluable, and which in time will be fully appreciated. Abbott has the confidence of the profession because he makes good. On his presentation it was promptly tried, and another success is scored.

---

For Sale.—One 10-plate Van Houten and Ten Broeck static machine with complete X-ray outfit. Good as new.—H. B. Hendrix, Cement, Okla.

---

The J. A. M. A. propaganda in favor of U. S. P. and N. F. pharmacal products as against the better class of proprietary pharmaceuticals, as well as the horde of what are usually grouped together as "nostrums," meaning preparations whose formulas are not published, although the products themselves are advertised only to the profession, in its last analysis throws the burden of production upon the large pharmaceutical houses, because the average competent pharmacist, while an able prescriptionist and, in the majority of cases, an honest man, by preference has too incomplete an equipment to enable him to make all of these products with the acquired accuracy, uniformity, elegance, standardization and yet without waste as by loss of alcohol, for example.

He may be willing and, indeed, able to assay his opium or his aconite as crude drugs and his fluid extracts of them as finished products, but has he, as a rule, the necessary apparatus with which to conduct this important work?

The U. S. P. demands the assay of everything assayable. Even if the retailer buys assayed opium and aconite and carefully makes up his fluid extracts of them, he must be able to standardize his finished products, else they will be U. S. P. only in name.

The propaganda in favor of what might be called "home-made pharmacy" would, therefore, defeat itself if all prescriptions for U. S. P. and N. F. products were to be filled only with the output of the retail pharmacist.

For many and obvious reasons a well-equipped laboratory on a large scale, manned by experts, backed by capital and experience and with a reputation for fair square dealing back of it all can with more exactness, greater despatch and that highly essential uniformity turn out a superior line of official products than could possibly be produced in a small way by even the most conscientious retailer.

It, therefore, logically follows that this is the real kernel of this much talked of nut, and this is why one of our old and conservative advertisers has taken this as the topic of his advertising chat this month.

---

PARMELE, a name already a household word with the medical profession of America, has still more and greatly endeared himself to the doctors by his self-sacrificing and successful labors in fighting that great evil, substituting "something just as good" in physicians' prescriptions. He is the author of the Page bill, now a law in New York, making substitution a felony, and he fought it through the Legislature almost single-handed, opposed by powerful influences—the combined money, strength and all sorts of underhanded tactics of well known substituters and grafters. Now let us take this New York law for the pattern, and get such an one through the Texas Legislature,—follow the brave and successful lead of "Parmele the Peerless," who has ever championed the rights of the medical men.

**YE EDITOR'S VACATION.**

(With apologies to Gilbert and Sullivan.)

---

In the shadow of the beeches on the bank,
By a cool and curling creeklet thro' the sand—
Budweiser's in the bucket, the ice is in the glass,
The coffee's on the embers, and the bacon's in the pan,
And Sam, the cook, is waiting, with his scaling knife in hand,
To serve us up a supper of this BIG, BLACK BASS!

TRACHOMA THREATENING TEXAS.—On account of the prevalence of trachoma among the numerous immigrants trying to get into Texas via Mexico, Dr. G. W. Storer, of the P. H. and M. H. Service, has been sent to Texas to assist and instruct local physicians who have to deal with them.

---

THE NEW STATE PHARMACY BOARD consists of Drs. W. H. Roberts, Jr., Denison; T. J. Snell, Cooper; W. P. Robertson, Gonzales; Jno. Weeks, Ballinger, and Bruce Verdenburg of Beaumant. The Board met at Austin August 27th, and elected Dr. Roberts President; Dr. Robertson, Treasurer, and Dr. R. H. Walker, of Gonzales, Secretary. Dr. Walker is the long-time secretary of the State Pharmacy Association, and is eligible though not a member of the Board. The first meeting to examine applicants for license will be held at Dallas, September 17th inst.

---

A STRAW.—Dr. E. S. McKee, in the *Therapeutic Record*, Louisville, Ky., says:

"The American Medical Editors held a very interesting convention for two days preceding the American Medical Association. They devoted probably too much time to denouncing the *Journal of the American Medical Association* and the State journals. * * * There was a regrettable tendency in the association to break away from the Americal Medical Association."

Another case of "*parturient montes.*" Not even a *ridiculus mus* of a resolution of protest against the tyranny and insolence of the Octopus was "brought forth." Will the "independent press" stand further kicks?

---

GRANULATED DRY BEEF ("Meatox") is the latest advance in concentrated foods. Prof. Ch. Marchand, Drevet Chemical Co., New York, has perfected his experiments begun in 1883, and now presents to the medical profession a granulated dry beef, free from preservatives, a food in its most concentrated form which will not deteriorate with age. I have tried it. It "tastes good," and will make an excellent luncheon, but it is admirably adapted to sick and convalescent. I give herewith an analysis by Prof. Tresh of the Public Health Laboratories of London. Prof. Marchand, who is chemist and graduate of the Central School of Arts and Manufactures of Paris, and whose New York address is 57 Prince Street, will send free on request full information about this valuable food.

THE EDITOR (he objects to being called "This here Jones") of the California Tentacle of the Octopus, has had something like the experience of the parrot—Polly was monkeying with a sleeping mastiff and came to grief. Slowly crawling up a tree, she dismally eyed her delapidated condition, and said: "I know what's the matter with me. I talk too damned much." Jonesey—"this here Jones"—after vilifying and denouncing as "unclean" and "infectious" all of the "individually-owned-and-conducted-for-profit-journals," as he calls the entire independent medical press, had, the, er—er—shall I say "gall," or assurance—or effrontery to apply for membership in the Association of American Medical Editors. He was given a lemon—on ice. I guess he realizes,—or will realize by the time he gets through with that suit for libel,—that, like the parrot, he talks too damned much.

---

DR. MOODY'S SANATORIUM, SAN ANTONIO.—Dr. Jno. A. McIntosh, for some years Assistant Superintendent of the Southwestern Texas Insane Asylum at San Antonio, has resigned that position to accept an appointment on the staff of the Moody Sanatorium. There are now three resident physicians at this well known institution who devote their whole time to it, and to the specialty of mental and nervous diseases. The pathological department is now admirably equipped and officered, and a thorough clinical study is made of each patient. The average daily population of patients runs from forty to sixty.

Dr. McIntosh graduated from the Medical Department of the University of Texas in 1903, and was immediately appointed pathologist of the John Sealy Hospital, Galveston. This position he held until November, 1903, when he was appointed by Governor Sayers Assistant Superintendent of the Southwestern Insane Asylum. In both these positions he gave most excellent and satisfactory service.

## Figures and Tables

**Figure f1:**